# Disparities in Postpartum Care Visits: The Dynamics of Parental Leave Duration and Postpartum Care Attendance

**DOI:** 10.1007/s10995-024-03929-z

**Published:** 2024-05-25

**Authors:** Brianna Keefe-Oates, Elizabeth Janiak, Barbara Gottlieb, Jarvis T. Chen

**Affiliations:** 1 Department of Social and Behavioral Sciences, Harvard School of Public Health, Boston, MA USA; 2 Department of Obstetrics and Gynecology, Brigham and Women’s Hospital, Boston, MA USA; 3 Department of Medicine, Harvard Medical School, Boston, MA USA

**Keywords:** Postpartum care, Health Policy, Parental Leave

## Abstract

**Objectives:**

To understand differences in the relationship between parental leave duration and postpartum care across sociodemographic and income groups.

**Methods:**

We used data from six states participating in the Center for Disease Control and Prevention’s yearly PRAMS study from 2016 to 2019 with a total sample of 12,442 people. Bivariable analyses assessed demographics among those who took more or less parental leave and estimated the prevalence of not accessing postpartum care by demographics, stratified by leave length. We used propensity score weighting to estimate the predicted risk and risk ratios of not accessing postpartum care with < 7 as compared to > = 7 weeks of leave, stratified by income.

**Results:**

There were significant differences in the prevalence of not accessing care stratified by leave duration, and disparities in utilization by race, ethnicity, and income. A shorter leave duration was associated with a higher risk of not accessing care (RR: 1.98 [CI 1.25–3.20] in higher income group, RR: 1.45 [CI 1.08, 1.99] in lower). The absolute risk of not accessing care was highest in the lower income group regardless of leave duration, though patterns of increased utilization with longer leave duration were consistent in both groups.

**Conclusions for practice:**

While shorter leave durations increased the risk of not attending postpartum care, those with lower incomes had the highest absolute risk of not attending care. Policies to support paid leave and extended leave duration are necessary, along with additional supports to increase postpartum care utilization, particularly among low-income families.

**Supplementary Information:**

The online version contains supplementary material available at 10.1007/s10995-024-03929-z.

## Significance

**What is already known on this subject?:**

Postpartum care helps identify pregnancy and birth-related complications and coordinate follow-up care, serving as an important avenue for identification and treatment of maternal morbidities. Parental leave is associated with improved health outcomes postpartum. Disparities in both parental leave and postpartum care have been observed across multiple sociodemographic groups.

**What does this study add?:**

A shorter leave duration increased the risk of not attending postpartum care. However, those with lower incomes had the highest absolute risk of not attending care. These findings suggest leave duration is important for postpartum care attendance, and that low-income families should receive further support to improve postpartum care attendance regardless of leave duration.

**Supplementary Information:**

The online version contains supplementary material available at 10.1007/s10995-024-03929-z.

## Introduction

The postpartum period, defined as the first 12 weeks after a delivery, can be an emotionally and physically challenging time for the birthing person who is often caring for a new baby while also recovering from pregnancy and childbirth. (Paladine et al., 2019) Postpartum healthcare visits, commonly offered by the provider who saw an individual during their pregnancy, are intended to be comprehensive assessments of a person’s physical and mental health postpartum.(Optimizing Postpartum Care, 2018). The visits serve multiple purposes, depending on the individual, and include monitoring physical and mental healthcare needs that developed or were exacerbated during pregnancy, labor, and delivery, and coordinating follow-up care. (Essien et al., 2019; Masho et al., 2016; Paladine et al., 2019; Stuebe et al., 2021; US Preventive Services Task Force, 2019) Though there is little documented research on the health outcomes of attending postpartum care at the population level, postpartum care has been associated with increased contraceptive use and improved screening and detection of postpartum depression.(Masho et al., 2016; Stuebe et al., 2021; US Preventive Services Task Force, 2019) Monitoring in the postpartum period is especially important given that in the United States, which has a higher maternal mortality rate than all other high income and many middle- and low-income countries, one fifth of maternal deaths occur between 7 and 42 days postpartum. (Centers for Disease Control and Prevention, 2020; Stuebe et al., 2021) The importance of this period is underscored by the fact that maternal mortality and morbidity rates in the United States are highly inequitable. Due to historical and present racial discrimination and a healthcare system in which people with higher incomes and better job benefits have better access to healthcare, Native and Black Americans experience disproportionately higher rates of maternal morbidity and mortality.(Bailey et al., 2017; Centers for Disease Control and Prevention, 2020).

Recognizing these inequities, the American College of Obstetricians and Gynecologists (ACOG) has recently changed their recommendations for postpartum care from a single check-up at 6 weeks postpartum to continuous contact with a healthcare provider in the first 12 weeks. (Optimizing Postpartum Care, 2018) Yet despite the updated recommendations, the proportion attending at least a single postpartum care appointment is only 72%.(Attanasio et al., 2022) Prior research has shown that barriers to postpartum care include a lack of transportation, childcare demands, time related to caring for a new baby, and work, family, and/or school responsibilities.(Henderson et al., 2016; Morgan et al., 2018; Rodin et al., 2019) Studies have found that people who are marginalized in the United States, including those who are younger, uninsured, lower income, and belong to minoritized racial or ethnic groups, are less likely to attend postpartum care because of greater vulnerability to the structural barriers listed above. (Danilack et al., 2019; DiBari et al., 2014; Morgan et al., 2018; Parekh et al., 2018; Thiel de Bocanegra et al., 2017; Wilcox et al., 2016)

For people who are employed in the postpartum period, their ability to take leave – whether paid or unpaid – and their length of leave, influence their ability to attend care. ​ The most recent research has found that approximately 66% of first-time mothers^1^ work during pregnancy in the United States, with 17% of all mothers returning to work within 6 weeks.(Horowitz et al., 2017) Being able to take enough time off of work to care for oneself has been shown to improve postpartum health outcomes: research has found that increased leave duration improves breastfeeding outcomes, while paid family leave policies, which increase leave duration, have been shown to improve postpartum health outcomes including decreased rates of hospitalizations, improved mental health, and increased breastfeeding initiation and duration.(Beuchert et al., 2016; Chai et al., 2018; Heshmati et al., 2023; Jou et al., 2018; Kortsmit et al., 2021) Yet no studies thus far have explored whether leave duration influences postpartum care attendance. ​

A longer parental leave could reduce barriers to attending postpartum care appointments. The length of leave a person takes is highly correlated with the amount of paid leave they are offered, as unpaid leaves are not sustainable for many parents. (Baum II & Ruhm, 2016) Yet paid parental leave for any amount of time is not guaranteed in the United States. The sole federal policy, the Family Medical Leave Act, only mandates large employers provide 12 weeks of unpaid leave. (Bipartisan Policy Center, 2022) Paid leave is mandated by some states and offered voluntarily by some employers. This varied policy landscape leads to inequitable patterns in leave and leave duration. While people working for larger companies with better benefits are often offered longer, paid leave, people working in other sectors are not. Historical and current social practices rooted in structural racism can restrict access to stable employment and jobs with generous benefits, including adequate parental leave. Inequitable access to employment benefits and the absence of national policy supporting paid leave has led to disparities in leave-taking, with those who receive paid leave being more likely to be white and earn a higher income (Goodman et al., 2021; Hawkins, 2020; Horowitz et al., 2017; KFF, 2021). Underscoring this inequity, a recent survey found that while the median leave duration for birthing parents was 11 weeks, the duration for employees with lower incomes was 6 weeks, coinciding with the usual timing of the postpartum visit.(Horowitz et al., 2017) Consequently, inequities in leave patterns may also exacerbate inequities in the ability to attend postpartum care.

As policymakers at the state and national levels are introducing paid family and medical leave laws for use after a birth or adoption of a new child, it is critical that these laws are designed to eliminate rather than reinforce existing inequities. (State Policies to Improve Maternal Health Outcomes, 2020) The specific policy provisions in each law, including how much leave is paid and the level at which it is paid, may determine how much leave an individual is able to take, which in turn could impact their ability to access healthcare.(Bipartisan Policy Center, 2022) Little research has focused on how leave duration, specifically, directly impacts postpartum care utilization in the US.(Steenland et al., 2021) The objectives of this study were to understand patterns of leave duration across sociodemographic groups, with a specific focus on income, and to assess how leave duration is associated with postpartum care in an environment without state or federal paid leave.

## Methods

We analyzed data from the Centers for Disease Control and Prevention’s (CDC) Pregnancy Risk Assessment Monitoring System (PRAMS) to describe: (a) patterns of postpartum care attendance among people working during their pregnancy, and (b) associations between leave length and postpartum care attendance stratified by income. We hypothesized that income may modify the relationship between leave-taking length and postpartum care attendance, as people who take less leave but have higher incomes may be more likely to have the resources to access care despite a short leave-taking period.

The PRAMS survey is a collaboration between the CDC and state and local health departments designed to produce state-level estimates of key pregnancy, postpartum, and infant health outcomes. Individuals who had a live birth in the calendar year are sampled from state birth certificate files and invited to participate via mail and telephone; respondents fill out the survey 2–6 months after giving birth. Depending on the state, the sampling strategy may feature oversampling by certain demographic and birth characteristics. Sample weights are provided to enable calculation of state and national-level estimates. (Shulman et al., 2018)

### Population

We included data from states that participated in PRAMS between 2016 and 2019 and whose surveys included questions regarding working status during pregnancy and leave-taking postpartum as well as relevant covariates. To mitigate potential confounding by recent policy changes, and to describe existing disparities without these policies, we excluded: (a) states with paid leave laws, and (b) states that changed duration of postpartum Medicaid coverage during these years. The final six states included in our analytic sample were: Maryland, Massachusetts, New Hampshire, North Carolina, Oregon, and Wisconsin.

We restricted the sample from each state to individuals who were employed during their pregnancy and returned to their jobs after their pregnancy or reported intending to return (if still on leave at the time of the survey), and were 18 years or older. We removed anyone reporting more than 52 weeks’ leave (less than 0.1% of the original six-state sample) as we suspected that this may be a data entry error, or that these rare experiences would be very different from most experiences working and attending postpartum care.

### Measures

The primary exposure was a shorter leave duration, defined as < 7 weeks of leave. We hypothesized that people who returned within or immediately after six weeks may not have the time to attend postpartum care, which is frequently scheduled six weeks after birth. Respondents reported how many weeks or months of leave they had or planned to take (if still on leave). All reporting in months was converted to number of weeks. The primary outcome was whether an individual reported attending a postpartum checkup.

To examine effect measure modification by income, we categorized individuals as earning above or below 200% of the 2019 Federal Poverty Limit (FPL). In the PRAMS survey respondents were asked their pre-tax household income in the 12 months prior to their baby’s birth and were provided categorical response options. We used this number and the number of people in the respondent’s household to calculate FPL using the US Health and Human Services poverty guidelines, which classify the percentage of the FPL based on household size and income (ASPE, 2024). In the PRAMS survey, the income category response options ranged from “$0-$16,000” through “$85,001 or more” with varying increments of between $3,000 and $13,000 for each category. We only categorized people into the ‘below 200% FPL’ category if their reported income and household size was included in the 200% FPL range of the HHS guidelines. If a respondent’s reported income category straddled the cutoff for 200% FPL after combining with their household size, they were classified into the ‘above 200% FPL’ category for a conservative estimate. We could not determine the FPL of families with 8 or more household members that earned more than $85,000 and thus excluded these respondents (< 0.1% of the total PRAMS sample).

We included relevant variables in our models that had a theoretical or empirical association with both postpartum care and leave-taking. These covariates included: age, marital status (married or not), educational attainment (Bachelor’s degree or more, compared to less), and number of previous live births (any or no previous birth). We also created variables for maternal race and ethnicity (proxies of experiencing racism and discrimination) reported on the baby’s birth certificate. We coded race as: “Asian-American, Hawaiian or Pacific Islander,” “Another race” (due to small sample sizes this included respondents reporting ‘American Indian/Alaska Native’, ‘Mixed Race’, or ‘Other race’), “Black,” and “White”. We created a separate binary variable for Hispanic and non-Hispanic individuals. We included two covariates which were self-reported on the survey. One was ‘current insurance payor’, with categories including ‘Medicaid’ (insurance for low-income families funded through state and federal governments), ‘Healthcare exchanges’ (private insurance for individuals that is not through their employer, and sometimes subsidized by the government), ‘Employer’ (insurance provided through an employer, though the employee often pays a portion), ‘Other’, and ‘None’ (See supplemental table for additional information). The second self-reported covariate was whether the respondent received any paid leave; unfortunately the survey did not collect data on the amount of paid leave. Models adjusting for covariates also included the respondent’s infant’s age in days (presented in Table 1 as age in weeks) and the language in which the survey was answered (Spanish or English).

**Table 1:** Demographic characteristics of individuals employed and returning to place of employment postpartum by length of leave

	Overall *n* = 12,442 % (CI)*	7 or more weeks *n* = 10,026 % (CI)*	Less than 7 weeks *n* = 2,416 % (CI)
*Household income*			
Above 200% FPL	71.5 (70.4, 72.7)	77.2 (76.0, 78.4)	48.0 (45.0, 51.0)
Below 200% FPL	28.5 (27.3, 29.6)	22.8 (21.6, 24.0)	52.0 (49.0, 55.0)
Missing	*n* = 473	*n* = 349	*n* = 124
*Bachelor’s degree or higher*			
Yes	55.5 (54.3, 56.7)	61.0 (59.6, 62.3)	33.3 (30.7, 36.0)
No	44.5 (43.3, 45.7)	39.0 (37.7, 40.4)	66.7 (64.0, 69.3)
Missing	*n* = 107	*n* = 88	*n* = 19
*Married*			
Yes	70.4 (69.3, 71.6)	75.1 (73.9, 76.3)	51.3 (48.4, 54.2)
No	29.6 (28.4, 30.7)	24.9 (23.7, 26.1)	48.7 (45.8, 51.6)
Missing	*n* = 3	*n* = 2	*n* = 1
*Age*			
18–24	12.5 (11.6, 13.4)	9.6 (8.8, 10.5)	24.1 (21.5, 26.9)
25–29	26.7 (25.6, 27.8)	26.0 (24.8, 27.2)	29.5 (26.9, 32.2)
30–34	37.0 (35.8, 38.1)	39.5 (38.2, 40.8)	26.7 (24.3, 29.3)
35–39	19.7 (18.7, 20.6)	20.6 (19.6, 21.7)	15.7 (13.7, 17.9)
40+	4.2 (3.7, 4.7)	4.2 (3.7, 4.8)	4.0 (3.1, 5.2)
Missing	*n* = 0	*n* = 0	*n* = 0
*Race*			
Asian, Native Hawaiian, or Pacific Islander	5.5 (5.1, 5.9)	5.6 (5.1, 6.0)	5.0 (4.2, 5.9)
Black	16.4 (15.4, 17.3)	15.1 (14.1, 16.1)	21.7 (19.2, 24.3)
White	72.2 (71.1, 73.2)	74.1 (73.0, 75.2)	64.2 (61.3, 66.9)
Another race	6.0 (5.5, 6.6)	5.2 (4.7, 5.8)	9.2 (7.7, 11.0)
Missing	*n* = 104	*n* = 77	*n* = 27
*Hispanic ethnicity*			
Hispanic	9.3 (8.7, 9.8)	8.5 (8.0, 9.1)	12.2 (10.6, 13.9)
Non-Hispanic	90.7 (90.2, 91.3)	91.5 (90.9, 92.0)	87.8 (86.1, 89.4)
Missing	*n* = 134	*n* = 111	*n* = 23
*Previous birth*			
Yes	56.0 (54.8, 57.2)	54.9 (53.6, 56.2)	60.4 (57.5, 63.3)
No	44.0 (42.8, 45.2)	45.1 (43.8, 46.4)	39.6 (36.7, 42.5)
Missing	*n* = 12	*n* = 11	*n* = 1
*Insurance type*			
Employer	68.1 (66.9, 69.2)	74.3 (73.1, 75.5)	42.5 (39.7, 45.4)
Medicaid (Government funded)	17.6 (16.6, 18.6)	13.8 (12.9, 14.8)	32.9 (30.1, 35.7)
Healthcare Exchanges	4.1 (3.6, 4.6)	3.4 (2.9, 3.9)	6.7 (5.4, 8.3)
Other	5.5 (5.0, 6.1)	4.8 (4.2, 5.4)	8.5 (7.0, 10.3)
None	4.8 (4.2, 5.4)	3.7 (3.1, 4.2)	9.4 (7.6, 11.5)
Missing	*n* = 116	*n* = 82	*n* = 34
*Leave length*			
1 to 6 weeks	15.7 (14.8, 16.6)	--	79.3 (76.9, 81.5)
13 to 24 weeks	19.7 (18.8, 20.6)	24.5 (23.4, 25.7)	--
7 to 12 weeks	58.6 (57.4, 59.8)	73.0 (71.9, 74.2)	--
More than 24 weeks	1.9 (1.6, 2.3)	2.4 (2.0, 2.9)	--
No leave	4.1 (3.6, 4.6)	--	20.7 (18.5, 23.1)
Missing	*n* = 0	*n* = 0	*n* = 0
*Number of weeks of leave*			
Mean (CI)	11.0 (10.9, 11.2)	12.8 (12.6, 12.9)	3.8 (3.7, 4.0)
Missing	*n* = 0	*n* = 0	*n* = 0
*Any paid leave*			
Yes	52.8 (51.6, 54.1)	58.7 (57.4, 60.1)	28.8 (26.3, 31.5)
No	47.2 (45.9, 48.4)	41.3 (39.9, 42.6)	71.2 (68.5, 73.7)
Missing	*n* = 2	*n* = 2	*n* = 0
*State*			
Massachusetts	24.4 (23.8, 25.0)	26.5 (25.7, 27.2)	16.0 (14.3, 17.9)
Maryland	19.9 (19.2, 20.5)	20.3 (19.5, 21.0)	18.3 (16.4, 20.3)
North Carolina	25.1 (24.2, 26.0)	23.2 (22.1, 24.2)	32.8 (29.9, 35.8)
New Hampshire	4.2 (4.1, 4.4)	4.4 (4.2, 4.6)	3.5 (3.0, 4.1)
Oregon	5.5 (5.2%, 5.8%)	5.6 (5.3%, 6.0%)	4.9 (4.1%, 5.7%)
Wisconsin	21.0 (20.3, 21.6)	20.1 (19.3, 20.9)	24.6 (22.3, 27.0)
Missing	*n* = 0	*n* = 0	*n* = 0
*Survey language*			
English	96.5 (96.1, 96.9)	97.3 (96.9, 97.6)	93.4 (91.8, 94.7)
Spanish	3.5 (3.1, 3.9)	2.7 (2.4, 3.1)	6.6 (5.3, 8.2)
Missing	*n* = 0	*n* = 0	*n* = 0
Baby’s age in weeks			
Mean (CI)	16.2 (16.1, 16.2)	16.1 (16.0, 16.1)	16.9 (16.5, 17.1)
Missing	*n* = 0	*n* = 0	*n* = 0
*Attended a postpartum visit*			
Yes	96.0 (95.6, 96.5)	96.9 (96.4, 97.3)	92.6 (91.1, 93.9)
No	4.0 (3.5, 4.4)	3.1 (2.7, 3.6)	7.4 (6.1, 8.9)
Missing	*n* = 48	*n* = 28	*n* = 20

*Point estimates and 95% confidence intervals used sample weights

### Statistical Analyses

We stratified all tabulations of survey-weighted sample characteristics by leave duration and report the prevalence of selected covariates as survey-weighted estimates in Table 1 as well as the missingness in each category. In Table 2 we present survey-weighted sample estimates of not attending postpartum care by sociodemographic characteristic and leave duration, after removing anyone who did not report whether or not they attended postpartum care. To assess the association between leave duration and postpartum care, we first estimated unadjusted risk ratios and predicted risks of not attending care using log-Poisson models with survey weights. We then used propensity score weighting to adjust for multiple covariates. We computed inverse-probability of treatment weights (IPTW) using the covariates listed above as well as state and year fixed effects, stratified by income, and multiplied these by the survey weights. We applied the weights in log-Poisson models to estimate adjusted risks and risk ratios of not accessing care with a short, as compared to long, leave duration. After removing missing observations in the outcome and stratification variables, a total of 11,969 individuals were included in the unadjusted models (96.2% of a total sample of 12,442) and a total of 11,925 individuals were included in the adjusted models (95.8% of the total sample, Table 3). We conducted all analyses in R (Vienna, Austria) using the “WeightIt” and “survey” packages.(Greifer, 2022; Lumley, 2022). As this was an analysis of secondary data, the Institutional Review Board of the Harvard TH Chan School of Public Health determined it was not human subjects research.

**Table 2:** Prevalence of not accessing postpartum care among individuals employed and returning to place of employment postpartum by demographics and employment benefits

	Overall	7 or more weeks	Less than 7 weeks
	*n* = 12,394	*n* = 9,998	*n* = 2,396
	% (CI)*	% (CI)*	% (CI)*
*Overall*	4.0 (3.5, 4.4)	3.1 (2.7, 3.6)	7.4 (6.1, 8.9)
*Household Income*			
Above 200% FPL	2.2 (1.9, 2.7)	1.90 (1.5, 2.3)	4.4 (3.0, 6.4)
Below 200% FPL	8.1 (6.0, 9.5)	7.0 (5.7, 8.5)	10.2 (8.0, 12.8)
*Bachelor’s degree or higher*			
Yes	1.9 (1.6, 2.4)	1.6 (1.2, 2.1)	4.5 (2.9, 7.1)
No	6.5 (5.6, 7.4)	5.5 (4.6, 6.5)	8.8 (7.1, 10.9)
*Married*			
Yes	2.5 (2.1. 2.9)	2.1 (1.7, 2.5)	4.8 (3.5, 6.5)
No	7.5 (6.4, 8.8)	6.3 (5.1, 7.7)	10.1 (7.9, 12.8)
*Age*			
18–24	7.1 (5.5, 9.1)	5.3 (3.6, 7.7)	10.1 (7.1, 14.0)
25–29	4.5 (3.6, 5.5)	3.3 (2.6, 4.3)	8.6 (6.1, 12.1)
30–34	2.8 (2.2, 3.5)	2.4 (1.8, 3.2)	4.9 (3.1, 7.4)
35–39	3.1 (2.4, 4.1)	2.9 (2.1, 3.9)	4.6 (2.6, 7.9)
40+	5.6 (3.6, 8.6)	4.7 (2.8, 7.7)	9.7 (4.2, 21.1)
*Race*			
Asian, Native Hawaiian, or Pacific Islander	6.1 (4.4, 8.4)	5.5 (3.7, 8.2)	8.6 (4.8, 15.0)
Black	6.0 (4.9, 7.5)	5.0 (3.8, 6.6)	9.0 (6.3, 12.5)
White	3.0 (2.5, 3.5)	2.3 (1.9, 2.9)	6.0 (4.5, 7.9)
Another race	7.7 (5.6, 10.6)	5.7 (3.8, 8.4)	12.5 (7.4, 20.3)
*Hispanic ethnicity*			
Hispanic	8.4 (6.9, 10.3)	7.7 (6.0, 9.9)	10.5 (7.2, 15.0)
Non-Hispanic	3.5 (3.1, 4.0)	2.7 (2.3, 3.2)	7.0 (5.6, 8.7)
*Previous birth*			
Yes	4.7 (4.1, 5.5)	3.7 (3.1, 4.4)	8.7 (7.0, 10.9)
No	3.0 (2.4, 3.6)	2.5 (1.9, 3.1)	5.3 (3.6, 7.8)
*Insurance type*			
Employer	2.0 (1.6, 2.4)	1.8 (1.5, 2.3)	3.0 (1.9, 4.7)
Medicaid (Government Funded)	8.0 (6.7, 9.6)	6.0 (4.7, 7.7)	11.5 (8.7, 14.9)
Healthcare Exchanges	7.7 (5.1, 11.4)	8.0 (5.0, 12.5)	7.1 (3.0, 15.8)
Other	5.7 (3.7, 8.5)	4.6 (2.7, 7.9)	8.0 (4.2, 14.7)
None	12.4 (8.8, 17.1)	12.3 (7.9, 18.7)	12.4 (7.3, 20.4)
*Any paid leave*			
Yes	2.5 (2.0, 3.0)	2.1 (1.6, 2.6)	6.0 (3.9, 9.0)
No	5.6 (4.9, 6.5)	4.6 (3.9, 5.6)	8.0 (6.4, 9.9)
*State*			
Massachusetts	4.1 (3.4, 4.9)	3.5 (2.8, 4.3)	7.9 (5.2, 11.9)
Maryland	5.2 (4.2, 6.4)	4.2 (3.2, 5.5)	9.9 (6.8, 13.8)
North Carolina	3.4 (2.4, 4.7)	2.7 (1.8, 4.1)	5.3 (3.1, 8.8)
New Hampshire	3.0 (2.1, 4.4)	2.1 (1.3, 3.3)	7.8 (4.3, 13.7)
Oregon	4.3 (3.1, 6.0)	3.8 (2.6, 5.6)	6.7 (3.6, 12.3)
Wisconsin	3.5 (2.6, 4.5)	2.1 (1.4, 3.1)	8.1 (5.7, 11.4)

**Table 3:** Association between length of leave and not accessing postpartum care, stratified by income

	*Survey weighted sample adjusted using propensity score weighting, estimating the Average Treatment Effect (* *n* * = 11,969)*							
	*Less than 200% FPL *(*n = 8,177)*^*a*^				*Above 200% FPL (* *n* * = 3,792)* ^*b*^			
	*Unadjusted*		*Adjusted*		*Unadjusted*		*Adjusted*	
	Risk (%, (CI))	Risk Ratio (CI)	Risk (%, (CI))*	Risk Ratio (CI)	Risk (%, (CI))	Risk Ratio (CI)	Risk (%, (CI))*	Risk Ratio (CI)
Less than 7 weeks	10.17 (8.02, 12.81)	1.45 (1.07, 1.96)	10.08 (7.73, 12.43)	1.46 (1.08, 1.99)	4.43 (3.03, 6.44)	2.33 (1.50, 3.51)	3.82 (2.18, 5.45)	1.98 (1.25, 3.20)
7 or more weeks	7.00 (5.72, 8.54)	1 (reference)	6.90 (5.48, 8.33)	1 (reference)	1.90 (1.54, 2.35)	1 (reference)	1.93 (1.53, 2.34)	1 (reference)

Models using propensity score weighting adjusted for age, marital status, educational attainment, number of previous live births, maternal race, maternal ethnicity, insurance payor at time of the survey, receipt of any paid leave, infant’s age, language the survey was answered in, and fixed effects were added for state and year

*Predicted based on the propensity score distribution

^a^ n in adjusted sample is 8,150

^b^ n in adjusted sample is 3,775

## Results

A total of 12,442 respondents were included in the sample; after survey weighting this represented a population of approximately 670,927 people who gave birth between 2016 and 2019 in the six states, were employed prior to birth, and returned or intended to return to their job after the birth. Of the weighted sample, 20% took < 7 weeks of leave. A majority took 7–12 weeks leave, with the average being 11 weeks (See Table 1).

A majority of the survey-weighted sample was above 200% FPL (72%, CI: 70.4–72.7), had a bachelor’s degree or higher (56%, CI: 54.3–56.7), reported their race as white on their baby’s birth certificate (72%, CI:71.1–73.2), and were non-Hispanic (91%, CI: 8.7–9.8). There were significant differences demographically between those who did and did not take ≥ 7 weeks of leave. Among those who took < 7 weeks a majority were below 200% FPL and had less than a bachelor’s degree, while the opposite was true in the ≥ 7 weeks group. Black respondents made up a significantly higher proportion of those taking < 7 weeks of leave (22%, CI:19.2–24.3) as compared to those with ≥ 7 weeks of leave (15%, CI: 14.1–16.1), as did those of Another race (9.2%, CI: 7.7–11.0 in the < 7 weeks group compared to 5.2%, CI:4.7–5.8, in the ≥ 7 weeks group). Hispanic respondents were also disproportionately represented in the < 7 weeks category (12.3%, CI: 10.6–13.9, of the < 7 weeks category were Hispanic, compared to 8.5%, CI: 8.0-9.1, in the ≥ 7 weeks category).

The prevalence of not accessing a postpartum care visit among this survey-weighted sample was 4.0% (CI: 3.5–4.4). The prevalence ranged from 1.9% (CI:1.6–2.4) to 12.4% (CI:8.8–17.1) when stratifying by select demographics (See Table 2). There were significant differences in the prevalence of not accessing care between those who took ≥ 7 weeks of leave (3.1%, CI: 2.7, 3.6), as compared to those who took < 7 weeks (7.4%, CI:6.1, 8.9). Those with lower incomes had a higher prevalence of not accessing care (8.1% in the below 200% FPL group vs. 2.2% in the above 200% FPL group). There were also clear racial and ethnic disparities overall and by leave strata, with Black, Asian, Hawaiian and Pacific Islander, and those reporting Another race, all having significantly higher prevalence of not accessing care compared to White individuals, and Hispanic individuals with significantly higher prevalence of not accessing care compared to non-Hispanic individuals. These trends in not accessing care by demographics were consistent across levels of leave duration, though not statistically significant in the < 7 weeks of leave stratum.

In the adjusted model using survey and inverse probability of treatment weights, taking < 7 weeks of leave was associated with a significantly higher risk of not accessing care in both income groups; in the higher income group it was associated with a 98% higher risk of not accessing postpartum care (RR: 1.98, CI: 1.25, 3.20), while the risk was 46% higher in the lower income group (RR: 1.45, CI: 1.08, 1.99). In the lower income group, the predicted risk of not accessing postpartum care when taking < 7 weeks of leave was 10.08% (CI:7.73–12.43) compared to 6.90% (CI: 5.48–8.33) in the group taking ≥ 7 weeks of leave. In the higher income group, the risk when taking < 7 weeks of leave was 3.82% (CI: 2.18–5.45) compared to 1.90% (CI: 1.54–2.35) when taking ≥ 7 weeks of leave. Though the risk ratios demonstrate that having a longer leave duration decreases the risk of not accessing care among all groups, the absolute risk of not accessing care was highest among the lower income group, regardless of leave duration. (Table 3)

## Discussion

During the postpartum period, employed individuals must navigate how to take time off to care for their families and themselves, including attending postpartum care visits which are crucial for monitoring an individuals’ health after pregnancy, labor, and delivery. Studies suggest that people desire more, not fewer, quality postpartum visits, however in this and other studies we see patterned inequities in accessing even a single visit.(Henderson et al., 2016; Paladine et al., 2019; Peahl et al., 2020) This study sought to understand patterns of postpartum care access and leave duration among employed individuals. Across income groups there was a significantly higher risk of not attending postpartum care when taking less than 7 weeks of leave, even in analyses that used IPTW to account for confounding and survey weights to yield population-level estimates. This finding confirmed our hypothesis that leave duration is associated with postpartum care utilization.

The strength of the association between leave duration and not accessing a postpartum visit was stronger among the higher income group, suggesting people in that group are more likely to benefit from a longer leave duration. Importantly, people with lower incomes had consistently higher predicted risks of not attending a postpartum visit regardless of their leave duration. This suggests that for those individuals, barriers to postpartum care may not be overcome solely by increasing the length of parental leave. The documented barriers to postpartum care such as transportation, childcare, and other work, family, and school responsibilities are often correlated with wealth.(Henderson et al., 2016; Morgan et al., 2018; Rodin et al., 2019) To address such barriers, paid family leave policies must at a minimum be designed to replace wages in full.

Workforce participation and occupational experiences are key determinants of health. Educational and employment discrimination have restricted the ability to earn higher incomes and receive workplace benefits, including paid leave and quality health insurance, particularly among racially minoritized groups. The larger percentage of Asian American, Hawaiian or Pacific Islander, Black and Hispanic individuals and those indicating Another race with both shorter leave durations and lower postpartum care attendance is notable and highlights racialized disparities, which have been found in other studies. Similar to these other studies, we also found higher percentages of people who were younger, did not have private insurance, were not married, and were lower income did not access postpartum care, and that most of these groups also had shorter leave durations. (Danilack et al., 2019; DiBari et al., 2014; Morgan et al., 2018; Thiel de Bocanegra et al., 2017; Wilcox et al., 2016). While we focus on one structural factor – leave duration – as a determinant of postpartum care access in this study, we recognize that the consistent inequities in postpartum care seen here likely reflect the interaction of multiple barriers to care, including healthcare systems barriers and others rooted in structural inequities including medical racism and financial challenges.(Davis, 2019; Treder et al., 2022).

We cannot definitively establish whether an intervention to offer more leave would improve postpartum care access; however, our findings echo prior literature that has demonstrated health benefits associated with a longer leave duration, which can result from the introduction of paid leave policies. (Beuchert et al., 2016; Chai et al., 2018; Courtin et al., 2022; Dagher et al., 2014; Heshmati et al., 2023; Kortsmit et al., 2021; Rossin, 2011; Rossin-Slater & Uniat, 2019; Shumbusho et al., 2020) Though few studies have focused on the outcome of postpartum care attendance related to parental leave, one study in Rhode Island found the introduction of a paid parental leave policy increased postpartum care attendance.(Steenland et al., 2021).

Mandating paid parental leave, separate from a measurable impact on health, is a reasonable overarching policy supporting equity. It is possible that paid family leave could also extend leave duration, in turn improving postpartum access. Our data suggest that changes in paid leave would be more effective as one piece of a larger policy agenda to improve access and uptake of postpartum care. These could include systems improvements in healthcare including scheduling challenges, transportation (i.e. using telehealth), ensuring expanded insurance coverage for postpartum visits, and improving quality of care with special attention to addressing the roots of medical racism (Johnston et al., 2021; Julian et al., 2020; Pereira et al., 2022). Additional policies outside the healthcare system, such as improving childcare and social support for postpartum individuals could also improve postpartum care access. Further qualitative and quantitative research may help determine how to structure leave policies as well as other governmental and healthcare systems practices in an equitable way that supports all families, and facilitates access to postpartum care.

This study must be interpreted in the context of several limitations. We chose to analyze experiences of people in states without paid parental leave policies, yet some cities within those states may have had local ordinances that mandate paid parental leave for their residents; in these cases, the effect of leave duration on postpartum care may be underestimated given that paid leave policies in some cities may decrease inequities in leave duration. The percentage of individuals reporting attending a visit was higher than has been found in other samples.(Attanasio et al., 2022) Though the PRAMS survey weights are designed to account for non-response and be representative statewide based on key measured variables, the method of recruitment via mail and telephone during the first few months postpartum may produce a bias in who is able to and chooses to respond. However, this bias, where individuals with more time and resources may be overrepresented, likely underestimates the impact of leave duration on postpartum care.

There may be some bias due to missingness in the income category; while only 3.8% of our observations were missing due to non-response in the income or household size categories (used to calculate the federal poverty limit), it is possible that responses to this question were not missing at random, and could have impacted the results. Finally, in the PRAMS methodology the maternal race, age, and ethnicity variables were extracted from the infants’ birth certificate data. Though these reports are likely accurate for age, the ways in which race and ethnicity are ascertained on birth certificates may vary by state and include some misclassification based on how the questions were asked and who answered the questions on the birth certificate in each state. The biases mentioned here could be addressed in future studies through quasi-experimental study designs, prospective data collection, and novel recruitment methods that could reach a wider population.

Finally, this study only focused on people working during pregnancy who returned to work. People who did not return to work may have chosen to leave their jobs to focus on parenting, or because they encountered significant barriers (such as a lack of paid leave, or employment discrimination) to returning to their jobs. If individuals who stopped working did so because of poor leave policies and/or because they could not financially afford to take time off, our sample may be biased towards more middle- and upper-income individuals and those who were offered more leave, underestimating how much income may influence the association between leave and postpartum care. In addition, we did not include people who did not work at all during pregnancy. These individuals may also need additional income supports to help sustain their families and attend to their own health in the postpartum period. Future studies could examine the needs of people who left their jobs during or after pregnancy, as well as those who did not work during pregnancy, to understand how these experiences impacted their ability to access postpartum care and how government policies may further support families in the postpartum period.

### Public Health Implications

Increasing the length of parental leave through policies such as paid family leave could increase workers’ postpartum care attendance. Policymakers and interventionists should also investigate and address sociodemographic inequities in care and provide additional support for low-income families to take longer parental leave or otherwise ensure adequate income postpartum. Clinicians and researchers can work to recognize barriers to postpartum care attendance in healthcare systems and develop flexible and patient-centered models of care to ensure workers can receive care regardless of if and when they return to work.

### Electronic Supplementary Material

Below is the link to the electronic supplementary material.

Supplementary Material 1

## Data Availability

The data used in this analysis are part of a data sharing agreement with the Centers of Disease Control and Prevention. To access these data investigators must contact the PRAMS team at the CDC.

## Footnotes

1 Throughout this manuscript we use gender-neutral language to refer to people who are postpartum, recognizing that not everyone who is pregnant identifies as a woman. However, when the study itself only refers to ‘women’ or ‘mothers’ and only sampled people who were identified as ‘women’ in the research, we use that language here to accurately describe that study population.

## Code Availability

The data were analyzed using R; code can be shared upon request.
