# Correction: Disparities in Postpartum Care Visits: The Dynamics of Parental Leave Duration and Postpartum Care Attendance

**DOI:** 10.1007/s10995-024-03968-6

**Published:** 2024-07-24

**Authors:** Brianna Keefe-Oates, Elizabeth Janiak, Barbara Gottlieb, Jarvis T. Chen

**Affiliations:** 1 Department of Social and Behavioral Sciences, Harvard School of Public Health, Boston, MA USA; 2 Department of Obstetrics and Gynecology, Brigham and Women’s Hospital, Boston, MA USA; 3 Department of Medicine, Harvard Medical School, Boston, MA USA

**Correction to: Maternal and Child Health Journal** 10.1007/s10995-024-03929-z

The original version of this article unfortunately contained error in Table header.

In Table 1, the n values at the header should have 3 digits after the decimal point instead the article was published incorrectly with two digits.

**Table 1:** Demographic characteristics of individuals employed and returning to place of employment postpartum by length of leave

	Overall n = 12,442 % (CI)*	7 or more weeks n = 10,026 % (CI)*	Less than 7 weeks n = 2,416 % (CI)
*Household income*			
Above 200% FPL	71.5 (70.4, 72.7)	77.2 (76.0, 78.4)	48.0 (45.0, 51.0)
Below 200% FPL	28.5 (27.3, 29.6)	22.8 (21.6, 24.0)	52.0 (49.0, 55.0)
Missing	n = 473	n = 349	n = 124
*Bachelor’s degree or higher*			
Yes	55.5 (54.3, 56.7)	61.0 (59.6, 62.3)	33.3 (30.7, 36.0)
No	44.5 (43.3, 45.7)	39.0 (37.7, 40.4)	66.7 (64.0, 69.3)
Missing	n = 107	n = 88	n = 19
*Married*			
Yes	70.4 (69.3, 71.6)	75.1 (73.9, 76.3)	51.3 (48.4, 54.2)
No	29.6 (28.4, 30.7)	24.9 (23.7, 26.1)	48.7 (45.8, 51.6)
Missing	n = 3	n = 2	n = 1
*Age*			
18–24	12.5 (11.6, 13.4)	9.6 (8.8, 10.5)	24.1 (21.5, 26.9)
25–29	26.7 (25.6, 27.8)	26.0 (24.8, 27.2)	29.5 (26.9, 32.2)
30–34	37.0 (35.8, 38.1)	39.5 (38.2, 40.8)	26.7 (24.3, 29.3)
35–39	19.7 (18.7, 20.6)	20.6 (19.6, 21.7)	15.7 (13.7, 17.9)
40+	4.2 (3.7, 4.7)	4.2 (3.7, 4.8)	4.0 (3.1, 5.2)
Missing	n = 0	n = 0	n = 0
*Race*			
Asian, Native Hawaiian, or Pacific Islander	5.5 (5.1, 5.9)	5.6 (5.1, 6.0)	5.0 (4.2, 5.9)
Black	16.4 (15.4, 17.3)	15.1 (14.1, 16.1)	21.7 (19.2, 24.3)
White	72.2 (71.1, 73.2)	74.1 (73.0, 75.2)	64.2 (61.3, 66.9)
Another race	6.0 (5.5, 6.6)	5.2 (4.7, 5.8)	9.2 (7.7, 11.0)
Missing	n = 104	n = 77	n = 27
*Hispanic ethnicity*			
Hispanic	9.3 (8.7, 9.8)	8.5 (8.0, 9.1)	12.2 (10.6, 13.9)
Non-Hispanic	90.7 (90.2, 91.3)	91.5 (90.9, 92.0)	87.8 (86.1, 89.4)
Missing	n = 134	n = 111	n = 23
*Previous birth*			
Yes	56.0 (54.8, 57.2)	54.9 (53.6, 56.2)	60.4 (57.5, 63.3)
No	44.0 (42.8, 45.2)	45.1 (43.8, 46.4)	39.6 (36.7, 42.5)
Missing	n = 12	n = 11	n = 1
*Insurance type*			
Employer	68.1 (66.9, 69.2)	74.3 (73.1, 75.5)	42.5 (39.7, 45.4)
Medicaid (Government funded)	17.6 (16.6, 18.6)	13.8 (12.9, 14.8)	32.9 (30.1, 35.7)
Healthcare exchanges	4.1 (3.6, 4.6)	3.4 (2.9, 3.9)	6.7 (5.4, 8.3)
Other	5.5 (5.0, 6.1)	4.8 (4.2, 5.4)	8.5 (7.0, 10.3)
None	4.8 (4.2, 5.4)	3.7 (3.1, 4.2)	9.4 (7.6, 11.5)
Missing	n = 116	n = 82	n = 34
*Leave length*			
1 to 6 weeks	15.7 (14.8, 16.6)	–	79.3 (76.9, 81.5)
13 to 24 weeks	19.7 (18.8, 20.6)	24.5 (23.4, 25.7)	–
7 to 12 weeks	58.6 (57.4, 59.8)	73.0 (71.9, 74.2)	–
More than 24 weeks	1.9 (1.6, 2.3)	2.4 (2.0, 2.9)	–
No leave	4.1 (3.6, 4.6)	–	20.7 (18.5, 23.1)
Missing	n = 0	n = 0	n = 0
*Number of weeks of leave*			
Mean (CI)	11.0 (10.9, 11.2)	12.8 (12.6, 12.9)	3.8 (3.7, 4.0)
Missing	n = 0	n = 0	n = 0
*Any paid leave*			
Yes	52.8 (51.6, 54.1)	58.7 (57.4, 60.1)	28.8 (26.3, 31.5)
No	47.2 (45.9, 48.4)	41.3 (39.9, 42.6)	71.2 (68.5, 73.7)
Missing	n = 2	n = 2	n = 0
*State*			
Massachusetts	24.4 (23.8, 25.0)	26.5 (25.7, 27.2)	16.0 (14.3, 17.9)
Maryland	19.9 (19.2, 20.5)	20.3 (19.5, 21.0)	18.3 (16.4, 20.3)
North Carolina	25.1 (24.2, 26.0)	23.2 (22.1, 24.2)	32.8 (29.9, 35.8)
New Hampshire	4.2 (4.1, 4.4)	4.4 (4.2, 4.6)	3.5 (3.0, 4.1)
Oregon	5.5 (5.2%, 5.8%)	5.6 (5.3%, 6.0%)	4.9 (4.1%, 5.7%)
Wisconsin	21.0 (20.3, 21.6)	20.1 (19.3, 20.9)	24.6 (22.3, 27.0)
Missing	n = 0	n = 0	n = 0
*Survey language*			
English	96.5 (96.1, 96.9)	97.3 (96.9, 97.6)	93.4 (91.8, 94.7)
Spanish	3.5 (3.1, 3.9)	2.7 (2.4, 3.1)	6.6 (5.3, 8.2)
Missing	n = 0	n = 0	n = 0
*Baby’s age in weeks*			
Mean (CI)	16.2 (16.1, 16.2)	16.1 (16.0, 16.1)	16.9 (16.5, 17.1)
Missing	n = 0	n = 0	n = 0
*Attended a postpartum visit*			
Yes	96.0 (95.6, 96.5)	96.9 (96.4, 97.3)	92.6 (91.1, 93.9)
No	4.0 (3.5, 4.4)	3.1 (2.7, 3.6)	7.4 (6.1, 8.9)
Missing	n = 48	n = 28	n = 20

*Point estimates and 95% confidence intervals used sample weights

The corrected table is presented here.

The original article has been corrected.

