# Supplementary material for: Disparities in Postpartum Care Visits: The Dynamics of Parental Leave Duration and Postpartum Care Attendance
Source: Matern Child Health J. 2024 May 25;28(9):1506–16. doi: 10.1007/s10995-024-03929-z (PMC11358175; doi:10.1007/s10995-024-03929-z)
Supplement: Supplementary file 1 — Supplementary Material 1 [file 10995_2024_3929_MOESM1_ESM.docx]

| **Supplemental Table 1. Association between length of leave and not accessing postpartum care, stratified by income, unweighted sample** | | | | | | | |  |
| --- | --- | --- | --- | --- | --- | --- | --- | --- |
|  | ***Unweighted sample adjusted using propensity score weighting and estimating the Average Treatment Effect (n = 11,969)*** | | | | | | |  |
|  | *Less than 200% FPL (n = 8,177)** | | | | *Above 200% FPL (n = 3,792)*** | | | |
|  | *Unadjusted* | | *Adjusted* | | *Unadjusted* | | *Adjusted* | |
|  | Risk (%) | Risk Ratio (CI) | Risk (%, (CI)) | Risk Ratio (CI) | Risk (%) | Risk Ratio (CI) | Risk (%, (CI)) | Risk Ratio (CI) |
| Less than 7 weeks | 9.54 | 1.41  (1.15, 1.73) | 10.94 (9.09, 12.80) | 1.31  (1.07, 1.62) | 5.40 | 2.20  (1.62, 2.94) | 4.18 (2.84, 5.72) | 1.72 (1.21, 2.48) |
| 7 or more weeks | 8.38 | 1  (reference) | 8.32 (7.20, 9.44) | 1  (reference) | 2.45 | 1  (reference) | 2.48 (2.11, 2.86) | 1  (reference) |
| *n in adjusted sample is 7,933 | | | |  |  |  |  |  |
| ** n in adjusted sample is 3,647 | | |  |  |  |  |  |  |

Supplemental Table 2. Health insurance survey questions and analysis categories

| **ORIGINAL QUESTION: What kind of health insurance do you have *now*?** Check ALL that apply | |
| --- | --- |
| *Original Response Option* | *Category for analysis* |
| Private health insurance from my job or the job of my husband or partner | Employer |
| Private health insurance from my parents | Employer |
| Private health insurance from the *< State >*Health Insurance Marketplace *or < statewebsite*>, or Healthcare.gov | Healthcare Exchanges |
| Medicaid (required: *state Medicaid name)* | Medicaid (Government Funded) |
| *State-specific option (Other government plan or program such as SCHIP/CHIP)* | Other |
| *State-specific option (Other government plan or program not listed above such as MCH program, indigent program or family planning program)* | Other |
| *State-specific option (TRICARE or other military health care)* | Other |
| *State-specific option (IHS or tribal)* | Other |
| Other health insurance: Please tell us: | Other |
| I do not have health insurance *now* | None |
